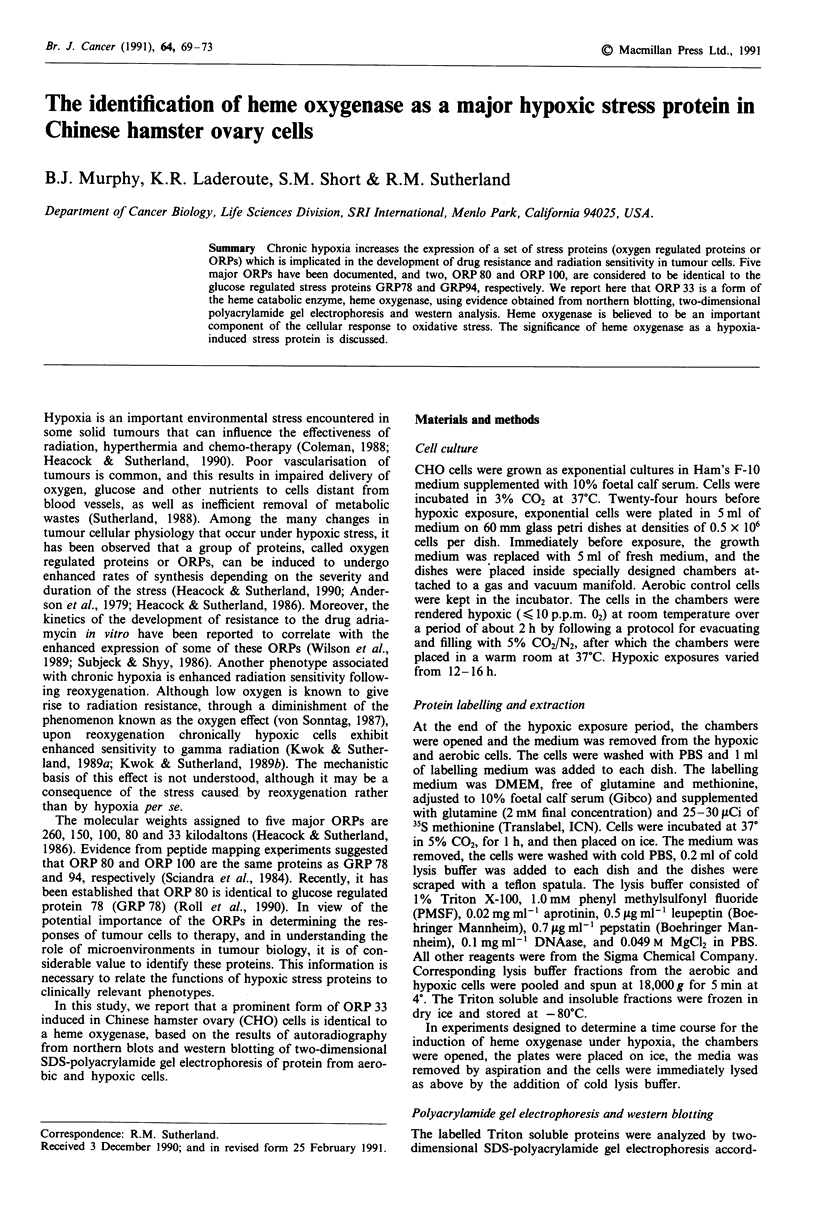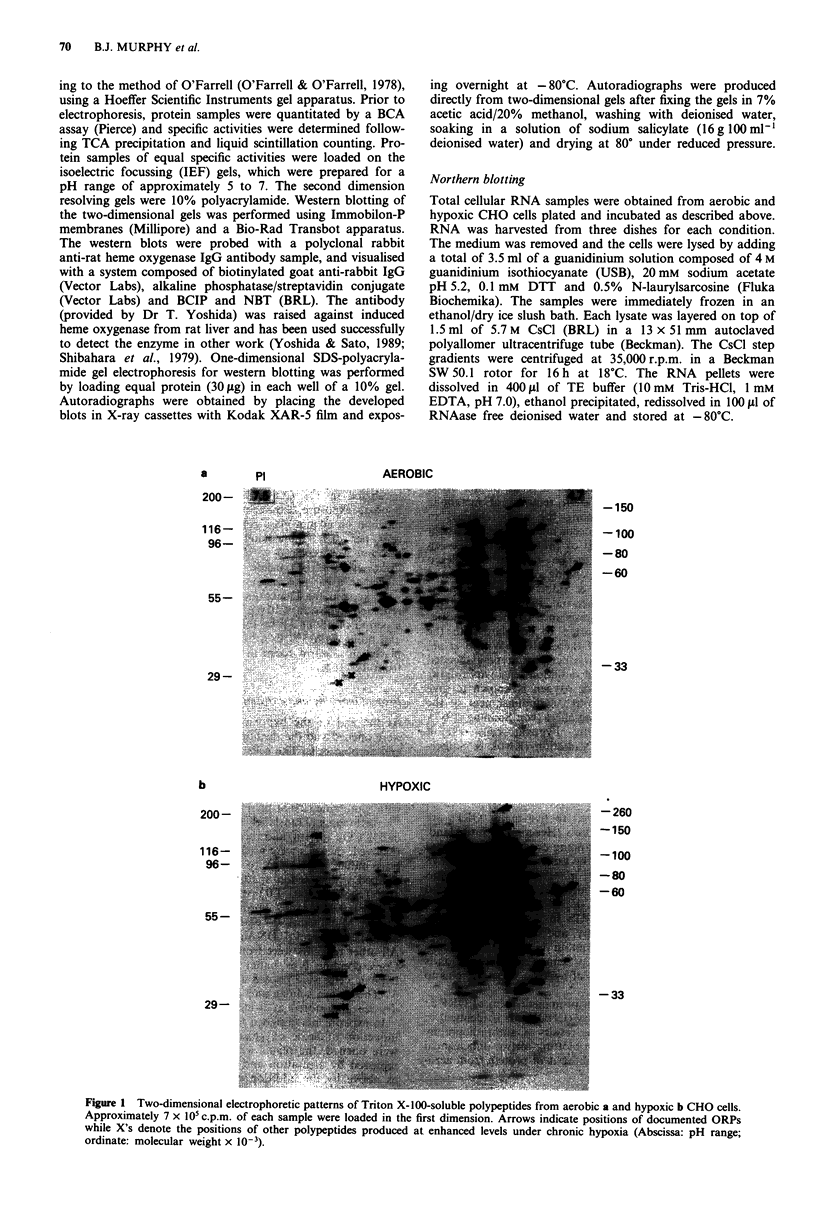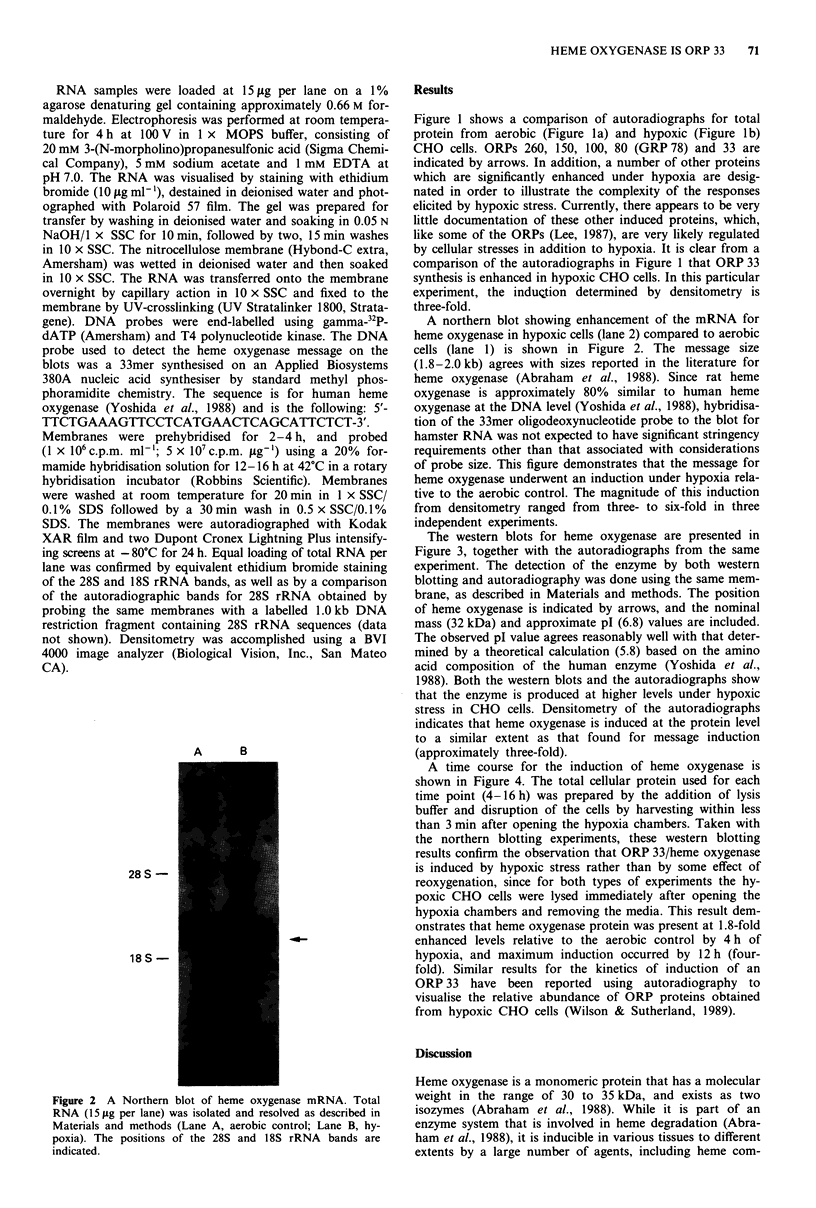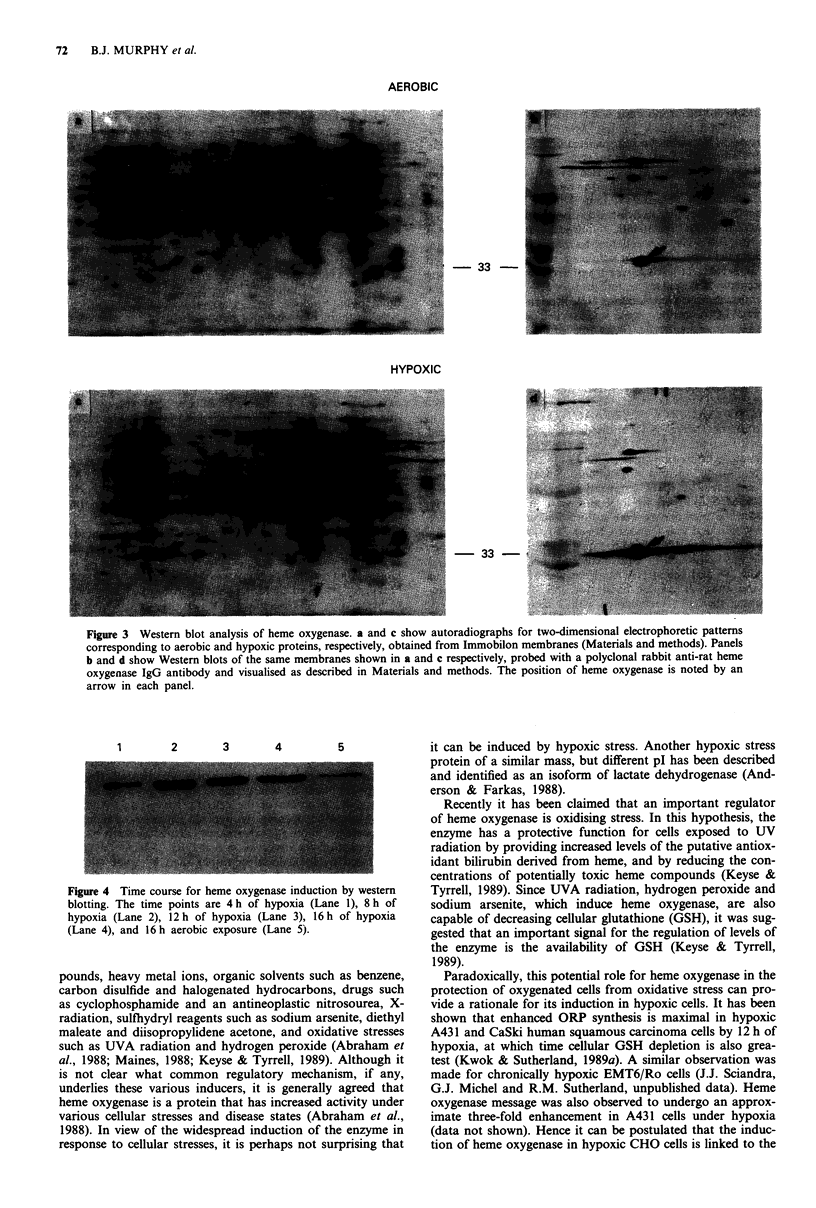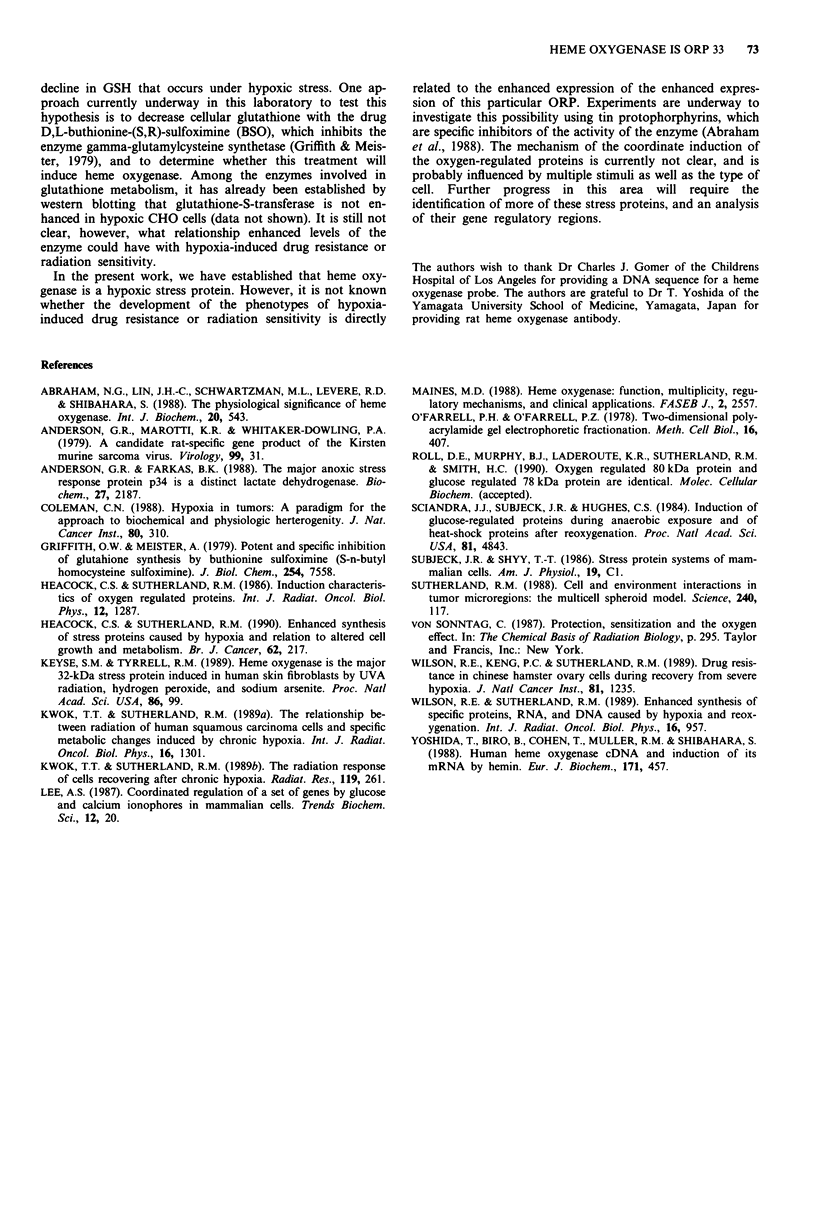# The identification of heme oxygenase as a major hypoxic stress protein in Chinese hamster ovary cells.

**DOI:** 10.1038/bjc.1991.241

**Published:** 1991-07

**Authors:** B. J. Murphy, K. R. Laderoute, S. M. Short, R. M. Sutherland

**Affiliations:** Department of Cancer Biology, SRI International, Menlo Park, California 94025.

## Abstract

**Images:**


Br. J. Cancer (1991), 64, 69–73 © Macmillan Press Ltd., 1991

### The identification of heme oxygenase as a major hypoxic stress protein in Chinese hamster ovary cells

B.J. Murphy, K.R. Laderoute, S.M. Short & R.M. Sutherland

*Department of Cancer Biology, Life Sciences Division, SRI International, Menlo Park, California 94025, USA.*

**Summary** Chronic hypoxia increases the expression of a set of stress proteins (oxygen regulated proteins or ORPs) which is implicated in the development of drug resistance and radiation sensitivity in tumour cells. Five major ORPs have been documented, and two, ORP 80 and ORP 100, are considered to be identical to the glucose regulated stress proteins GRP78 and GRP94, respectively. We report here that ORP 33 is a form of the heme catabolic enzyme, heme oxygenase, using evidence obtained from northern blotting, two-dimensional polyacrylamide gel electrophoresis and western analysis. Heme oxygenase is believed to be an important component of the cellular response to oxidative stress. The significance of heme oxygenase as a hypoxia-induced stress protein is discussed.

Hypoxia is an important environmental stress encountered in some solid tumours that can influence the effectiveness of radiation, hyperthermia and chemo-therapy (Coleman, 1988; Heacock & Sutherland, 1990). Poor vascularisation of tumours is common, and this results in impaired delivery of oxygen, glucose and other nutrients to cells distant from blood vessels, as well as inefficient removal of metabolic wastes (Sutherland, 1988). Among the many changes in tumour cellular physiology that occur under hypoxic stress, it has been observed that a group of proteins, called oxygen regulated proteins or ORPs, can be induced to undergo enhanced rates of synthesis depending on the severity and duration of the stress (Heacock & Sutherland, 1990; Anderson *et al.*, 1979; Heacock & Sutherland, 1986). Moreover, the kinetics of the development of resistance to the drug adriamycin *in vitro* have been reported to correlate with the enhanced expression of some of these ORPs (Wilson *et al.*, 1989; Subjeck & Shyy, 1986). Another phenotype associated with chronic hypoxia is enhanced radiation sensitivity following reoxygenation. Although low oxygen is known to give rise to radiation resistance, through a diminishment of the phenomenon known as the oxygen effect (von Sonntag, 1987), upon reoxygenation chronically hypoxic cells exhibit enhanced sensitivity to gamma radiation (Kwok & Sutherland, 1989*a*; Kwok & Sutherland, 1989*b*). The mechanistic basis of this effect is not understood, although it may be a consequence of the stress caused by reoxygenation rather than by hypoxia *per se*.

The molecular weights assigned to five major ORPs are 260, 150, 100, 80 and 33 kilodaltons (Heacock & Sutherland, 1986). Evidence from peptide mapping experiments suggested that ORP 80 and ORP 100 are the same proteins as GRP 78 and 94, respectively (Sciandra *et al.*, 1984). Recently, it has been established that ORP 80 is identical to glucose regulated protein 78 (GRP 78) (Roll *et al.*, 1990). In view of the potential importance of the ORPs in determining the responses of tumour cells to therapy, and in understanding the role of microenvironments in tumour biology, it is of considerable value to identify these proteins. This information is necessary to relate the functions of hypoxic stress proteins to clinically relevant phenotypes.

In this study, we report that a prominent form of ORP 33 induced in Chinese hamster ovary (CHO) cells is identical to a heme oxygenase, based on the results of autoradiography from northern blots and western blotting of two-dimensional SDS-polyacrylamide gel electrophoresis of protein from aerobic and hypoxic cells.

Correspondence: R.M. Sutherland.
Received 3 December 1990; and in revised form 25 February 1991.

### Materials and methods

### *Cell culture*

CHO cells were grown as exponential cultures in Ham's F-10 medium supplemented with 10% foetal calf serum. Cells were incubated in 3% $CO_2$ at 37°C. Twenty-four hours before hypoxic exposure, exponential cells were plated in 5 ml of medium on 60 mm glass petri dishes at densities of $0.5 \times 10^6$ cells per dish. Immediately before exposure, the growth medium was replaced with 5 ml of fresh medium, and the dishes were placed inside specially designed chambers attached to a gas and vacuum manifold. Aerobic control cells were kept in the incubator. The cells in the chambers were rendered hypoxic ($\leqslant$ 10 p.p.m. $0_2$) at room temperature over a period of about 2 h by following a protocol for evacuating and filling with 5% $CO_2/N_2$, after which the chambers were placed in a warm room at 37°C. Hypoxic exposures varied from 12–16 h.

### *Protein labelling and extraction*

At the end of the hypoxic exposure period, the chambers were opened and the medium was removed from the hypoxic and aerobic cells. The cells were washed with PBS and 1 ml of labelling medium was added to each dish. The labelling medium was DMEM, free of glutamine and methionine, adjusted to 10% foetal calf serum (Gibco) and supplemented with glutamine (2 mM final concentration) and 25–30 $\mu$Ci of $^{35}$S methionine (Translabel, ICN). Cells were incubated at 37° in 5% $CO_2$, for 1 h, and then placed on ice. The medium was removed, the cells were washed with cold PBS, 0.2 ml of cold lysis buffer was added to each dish and the dishes were scraped with a teflon spatula. The lysis buffer consisted of 1% Triton X-100, 1.0 mM phenyl methylsulfonyl fluoride (PMSF), 0.02 mg ml$^{-1}$ aprotinin, 0.5 $\mu$g ml$^{-1}$ leupeptin (Boehringer Mannheim), 0.7 $\mu$g ml$^{-1}$ pepstatin (Boehringer Mannheim), 0.1 mg ml$^{-1}$ DNAase, and 0.049 M $MgCl_2$ in PBS. All other reagents were from the Sigma Chemical Company. Corresponding lysis buffer fractions from the aerobic and hypoxic cells were pooled and spun at 18,000 *g* for 5 min at 4°. The Triton soluble and insoluble fractions were frozen in dry ice and stored at −80°C.

In experiments designed to determine a time course for the induction of heme oxygenase under hypoxia, the chambers were opened, the plates were placed on ice, the media was removed by aspiration and the cells were immediately lysed as above by the addition of cold lysis buffer.

### *Polyacrylamide gel electrophoresis and western blotting*

The labelled Triton soluble proteins were analyzed by two-dimensional SDS-polyacrylamide gel electrophoresis according to the method of O'Farrell (O'Farrell & O'Farrell, 1978), using a Hoeffer Scientific Instruments gel apparatus. Prior to electrophoresis, protein samples were quantitated by a BCA assay (Pierce) and specific activities were determined following TCA precipitation and liquid scintillation counting. Protein samples of equal specific activities were loaded on the isoelectric focussing (IEF) gels, which were prepared for a pH range of approximately 5 to 7. The second dimension resolving gels were 10% polyacrylamide. Western blotting of the two-dimensional gels was performed using Immobilon-P membranes (Millipore) and a Bio-Rad Transbot apparatus. The western blots were probed with a polyclonal rabbit anti-rat heme oxygenase IgG antibody sample, and visualised with a system composed of biotinylated goat anti-rabbit IgG (Vector Labs), alkaline phosphatase/streptavidin conjugate (Vector Labs) and BCIP and NBT (BRL). The antibody (provided by Dr T. Yoshida) was raised against induced heme oxygenase from rat liver and has been used successfully to detect the enzyme in other work (Yoshida & Sato, 1989; Shibahara *et al.*, 1979). One-dimensional SDS-polyacrylamide gel electrophoresis for western blotting was performed by loading equal protein (30 µg) in each well of a 10% gel. Autoradiographs were obtained by placing the developed blots in X-ray cassettes with Kodak XAR-5 film and exposing overnight at −80°C. Autoradiographs were produced directly from two-dimensional gels after fixing the gels in 7% acetic acid/20% methanol, washing with deionised water, soaking in a solution of sodium salicylate (16 g 100 $ml^{-1}$ deionised water) and drying at 80° under reduced pressure.

*Northern blotting*

Total cellular RNA samples were obtained from aerobic and hypoxic CHO cells plated and incubated as described above. RNA was harvested from three dishes for each condition. The medium was removed and the cells were lysed by adding a total of 3.5 ml of a guanidinium solution composed of 4 M guanidinium isothiocyanate (USB), 20 mM sodium acetate pH 5.2, 0.1 mM DTT and 0.5% N-laurylsarcosine (Fluka Biochemika). The samples were immediately frozen in an ethanol/dry ice slush bath. Each lysate was layered on top of 1.5 ml of 5.7 M CsCl (BRL) in a 13 × 51 mm autoclaved polyallomer ultracentrifuge tube (Beckman). The CsCl step gradients were centrifuged at 35,000 r.p.m. in a Beckman SW 50.1 rotor for 16 h at 18°C. The RNA pellets were dissolved in 400 µl of TE buffer (10 mM Tris-HCl, 1 mM EDTA, pH 7.0), ethanol precipitated, redissolved in 100 µl of RNAase free deionised water and stored at −80°C.

**Figure 1** Two-dimensional electrophoretic patterns of Triton X-100-soluble polypeptides from aerobic **a** and hypoxic **b** CHO cells. Approximately $7 \times 10^5$ c.p.m. of each sample were loaded in the first dimension. Arrows indicate positions of documented ORPs while X's denote the positions of other polypeptides produced at enhanced levels under chronic hypoxia (Abscissa: pH range; ordinate: molecular weight × $10^{-3}$).

RNA samples were loaded at 15 μg per lane on a 1% agarose denaturing gel containing approximately 0.66 M formaldehyde. Electrophoresis was performed at room temperature for 4 h at 100 V in 1 × MOPS buffer, consisting of 20 mM 3-(N-morpholino)propanesulfonic acid (Sigma Chemical Company), 5 mM sodium acetate and 1 mM EDTA at pH 7.0. The RNA was visualised by staining with ethidium bromide (10 μg $ml^{-1}$), destained in deionised water and photographed with Polaroid 57 film. The gel was prepared for transfer by washing in deionised water and soaking in 0.05 N NaOH/1 × SSC for 10 min, followed by two, 15 min washes in 10 × SSC. The nitrocellulose membrane (Hybond-C extra, Amersham) was wetted in deionised water and then soaked in 10 × SSC. The RNA was transferred onto the membrane overnight by capillary action in 10 × SSC and fixed to the membrane by UV-crosslinking (UV Stratalinker 1800, Stratagene). DNA probes were end-labelled using gamma-$^{32}$P-dATP (Amersham) and T4 polynucleotide kinase. The DNA probe used to detect the heme oxygenase message on the blots was a 33mer synthesised on an Applied Biosystems 380A nucleic acid synthesiser by standard methyl phosphoramidite chemistry. The sequence is for human heme oxygenase (Yoshida *et al.*, 1988) and is the following: 5'-TTCTGAAAGTTCCTCATGAACTCAGCATTCTCT-3'. Membranes were prehybridised for 2–4 h, and probed ($1 \times 10^6$ c.p.m. $ml^{-1}$; $5 \times 10^7$ c.p.m. $\mu g^{-1}$) using a 20% formamide hybridisation solution for 12–16 h at 42°C in a rotary hybridisation incubator (Robbins Scientific). Membranes were washed at room temperature for 20 min in 1 × SSC/0.1% SDS followed by a 30 min wash in 0.5 × SSC/0.1% SDS. The membranes were autoradiographed with Kodak XAR film and two Dupont Cronex Lightning Plus intensifying screens at −80°C for 24 h. Equal loading of total RNA per lane was confirmed by equivalent ethidium bromide staining of the 28S and 18S rRNA bands, as well as by a comparison of the autoradiographic bands for 28S rRNA obtained by probing the same membranes with a labelled 1.0 kb DNA restriction fragment containing 28S rRNA sequences (data not shown). Densitometry was accomplished using a BVI 4000 image analyzer (Biological Vision, Inc., San Mateo CA).

**Figure 2** A Northern blot of heme oxygenase mRNA. Total RNA (15 μg per lane) was isolated and resolved as described in Materials and methods (Lane A, aerobic control; Lane B, hypoxia). The positions of the 28S and 18S rRNA bands are indicated.

### Results

Figure 1 shows a comparison of autoradiographs for total protein from aerobic (Figure 1a) and hypoxic (Figure 1b) CHO cells. ORPs 260, 150, 100, 80 (GRP 78) and 33 are indicated by arrows. In addition, a number of other proteins which are significantly enhanced under hypoxia are designated in order to illustrate the complexity of the responses elicited by hypoxic stress. Currently, there appears to be very little documentation of these other induced proteins, which, like some of the ORPs (Lee, 1987), are very likely regulated by cellular stresses in addition to hypoxia. It is clear from a comparison of the autoradiographs in Figure 1 that ORP 33 synthesis is enhanced in hypoxic CHO cells. In this particular experiment, the induction determined by densitometry is three-fold.

A northern blot showing enhancement of the mRNA for heme oxygenase in hypoxic cells (lane 2) compared to aerobic cells (lane 1) is shown in Figure 2. The message size (1.8–2.0 kb) agrees with sizes reported in the literature for heme oxygenase (Abraham *et al.*, 1988). Since rat heme oxygenase is approximately 80% similar to human heme oxygenase at the DNA level (Yoshida *et al.*, 1988), hybridisation of the 33mer oligodeoxynucleotide probe to the blot for hamster RNA was not expected to have significant stringency requirements other than that associated with considerations of probe size. This figure demonstrates that the message for heme oxygenase underwent an induction under hypoxia relative to the aerobic control. The magnitude of this induction from densitometry ranged from three- to six-fold in three independent experiments.

The western blots for heme oxygenase are presented in Figure 3, together with the autoradiographs from the same experiment. The detection of the enzyme by both western blotting and autoradiography was done using the same membrane, as described in Materials and methods. The position of heme oxygenase is indicated by arrows, and the nominal mass (32 kDa) and approximate pI (6.8) values are included. The observed pI value agrees reasonably well with that determined by a theoretical calculation (5.8) based on the amino acid composition of the human enzyme (Yoshida *et al.*, 1988). Both the western blots and the autoradiographs show that the enzyme is produced at higher levels under hypoxic stress in CHO cells. Densitometry of the autoradiographs indicates that heme oxygenase is induced at the protein level to a similar extent as that found for message induction (approximately three-fold).

A time course for the induction of heme oxygenase is shown in Figure 4. The total cellular protein used for each time point (4–16 h) was prepared by the addition of lysis buffer and disruption of the cells by harvesting within less than 3 min after opening the hypoxia chambers. Taken with the northern blotting experiments, these western blotting results confirm the observation that ORP 33/heme oxygenase is induced by hypoxic stress rather than by some effect of reoxygenation, since for both types of experiments the hypoxic CHO cells were lysed immediately after opening the hypoxia chambers and removing the media. This result demonstrates that heme oxygenase protein was present at 1.8-fold enhanced levels relative to the aerobic control by 4 h of hypoxia, and maximum induction occurred by 12 h (four-fold). Similar results for the kinetics of induction of an ORP 33 have been reported using autoradiography to visualise the relative abundance of ORP proteins obtained from hypoxic CHO cells (Wilson & Sutherland, 1989).

### Discussion

Heme oxygenase is a monomeric protein that has a molecular weight in the range of 30 to 35 kDa, and exists as two isozymes (Abraham *et al.*, 1988). While it is part of an enzyme system that is involved in heme degradation (Abraham *et al.*, 1988), it is inducible in various tissues to different extents by a large number of agents, including heme com-

**Figure 3** Western blot analysis of heme oxygenase. **a** and **c** show autoradiographs for two-dimensional electrophoretic patterns corresponding to aerobic and hypoxic proteins, respectively, obtained from Immobilon membranes (Materials and methods). Panels **b** and **d** show Western blots of the same membranes shown in **a** and **c** respectively, probed with a polyclonal rabbit anti-rat heme oxygenase IgG antibody and visualised as described in Materials and methods. The position of heme oxygenase is noted by an arrow in each panel.

**Figure 4** Time course for heme oxygenase induction by western blotting. The time points are 4 h of hypoxia (Lane 1), 8 h of hypoxia (Lane 2), 12 h of hypoxia (Lane 3), 16 h of hypoxia (Lane 4), and 16 h aerobic exposure (Lane 5).

pounds, heavy metal ions, organic solvents such as benzene, carbon disulfide and halogenated hydrocarbons, drugs such as cyclophosphamide and an antineoplastic nitrosourea, X-radiation, sulfhydryl reagents such as sodium arsenite, diethyl maleate and diisopropylidene acetone, and oxidative stresses such as UVA radiation and hydrogen peroxide (Abraham *et al.*, 1988; Maines, 1988; Keyse & Tyrrell, 1989). Although it is not clear what common regulatory mechanism, if any, underlies these various inducers, it is generally agreed that heme oxygenase is a protein that has increased activity under various cellular stresses and disease states (Abraham *et al.*, 1988). In view of the widespread induction of the enzyme in response to cellular stresses, it is perhaps not surprising that it can be induced by hypoxic stress. Another hypoxic stress protein of a similar mass, but different pI has been described and identified as an isoform of lactate dehydrogenase (Anderson & Farkas, 1988).

Recently it has been claimed that an important regulator of heme oxygenase is oxidising stress. In this hypothesis, the enzyme has a protective function for cells exposed to UV radiation by providing increased levels of the putative antioxidant bilirubin derived from heme, and by reducing the concentrations of potentially toxic heme compounds (Keyse & Tyrrell, 1989). Since UVA radiation, hydrogen peroxide and sodium arsenite, which induce heme oxygenase, are also capable of decreasing cellular glutathione (GSH), it was suggested that an important signal for the regulation of levels of the enzyme is the availability of GSH (Keyse & Tyrrell, 1989).

Paradoxically, this potential role for heme oxygenase in the protection of oxygenated cells from oxidative stress can provide a rationale for its induction in hypoxic cells. It has been shown that enhanced ORP synthesis is maximal in hypoxic A431 and CaSki human squamous carcinoma cells by 12 h of hypoxia, at which time cellular GSH depletion is also greatest (Kwok & Sutherland, 1989*a*). A similar observation was made for chronically hypoxic EMT6/Ro cells (J.J. Sciandra, G.J. Michel and R.M. Sutherland, unpublished data). Heme oxygenase message was also observed to undergo an approximate three-fold enhancement in A431 cells under hypoxia (data not shown). Hence it can be postulated that the induction of heme oxygenase in hypoxic CHO cells is linked to the decline in GSH that occurs under hypoxic stress. One approach currently underway in this laboratory to test this hypothesis is to decrease cellular glutathione with the drug D,L-buthionine-(S,R)-sulfoximine (BSO), which inhibits the enzyme gamma-glutamylcysteine synthetase (Griffith & Meister, 1979), and to determine whether this treatment will induce heme oxygenase. Among the enzymes involved in glutathione metabolism, it has already been established by western blotting that glutathione-S-transferase is not enhanced in hypoxic CHO cells (data not shown). It is still not clear, however, what relationship enhanced levels of the enzyme could have with hypoxia-induced drug resistance or radiation sensitivity.

In the present work, we have established that heme oxygenase is a hypoxic stress protein. However, it is not known whether the development of the phenotypes of hypoxia-induced drug resistance or radiation sensitivity is directly related to the enhanced expression of the enhanced expression of this particular ORP. Experiments are underway to investigate this possibility using tin protophorphyrins, which are specific inhibitors of the activity of the enzyme (Abraham *et al.*, 1988). The mechanism of the coordinate induction of the oxygen-regulated proteins is currently not clear, and is probably influenced by multiple stimuli as well as the type of cell. Further progress in this area will require the identification of more of these stress proteins, and an analysis of their gene regulatory regions.

The authors wish to thank Dr Charles J. Gomer of the Childrens Hospital of Los Angeles for providing a DNA sequence for a heme oxygenase probe. The authors are grateful to Dr T. Yoshida of the Yamagata University School of Medicine, Yamagata, Japan for providing rat heme oxygenase antibody.

### References

ABRAHAM, N.G., LIN, J.H.-C., SCHWARTZMAN, M.L., LEVERE, R.D. & SHIBAHARA, S. (1988). The physiological significance of heme oxygenase. *Int. J. Biochem.*, **20**, 543.

ANDERSON, G.R., MAROTTI, K.R. & WHITAKER-DOWLING, P.A. (1979). A candidate rat-specific gene product of the Kirsten murine sarcoma virus. *Virology*, **99**, 31.

ANDERSON, G.R. & FARKAS, B.K. (1988). The major anoxic stress response protein p34 is a distinct lactate dehydrogenase. *Biochem.*, **27**, 2187.

COLEMAN, C.N. (1988). Hypoxia in tumors: A paradigm for the approach to biochemical and physiologic herterogenity. *J. Nat. Cancer Inst.*, **80**, 310.

GRIFFITH, O.W. & MEISTER, A. (1979). Potent and specific inhibition of glutahione synthesis by buthionine sulfoximine (S-n-butyl homocysteine sulfoximine). *J. Biol. Chem.*, **254**, 7558.

HEACOCK, C.S. & SUTHERLAND, R.M. (1986). Induction characteristics of oxygen regulated proteins. *Int. J. Radiat. Oncol. Biol. Phys.*, **12**, 1287.

HEACOCK, C.S. & SUTHERLAND, R.M. (1990). Enhanced synthesis of stress proteins caused by hypoxia and relation to altered cell growth and metabolism. *Br. J. Cancer*, **62**, 217.

KEYSE, S.M. & TYRRELL, R.M. (1989). Heme oxygenase is the major 32-kDa stress protein induced in human skin fibroblasts by UVA radiation, hydrogen peroxide, and sodium arsenite. *Proc. Natl Acad. Sci. USA*, **86**, 99.

KWOK, T.T. & SUTHERLAND, R.M. (1989*a*). The relationship between radiation of human squamous carcinoma cells and specific metabolic changes induced by chronic hypoxia. *Int. J. Radiat. Oncol. Biol. Phys.*, **16**, 1301.

KWOK, T.T. & SUTHERLAND, R.M. (1989*b*). The radiation response of cells recovering after chronic hypoxia. *Radiat. Res.*, **119**, 261.

LEE, A.S. (1987). Coordinated regulation of a set of genes by glucose and calcium ionophores in mammalian cells. *Trends Biochem. Sci.*, **12**, 20.

MAINES, M.D. (1988). Heme oxygenase: function, multiplicity, regulatory mechanisms, and clinical applications. *FASEB J.*, **2**, 2557.

O'FARRELL, P.H. & O'FARRELL, P.Z. (1978). Two-dimensional polyacrylamide gel electrophoretic fractionation. *Meth. Cell Biol.*, **16**, 407.

ROLL, D.E., MURPHY, B.J., LADEROUTE, K.R., SUTHERLAND, R.M. & SMITH, H.C. (1990). Oxygen regulated 80 kDa protein and glucose regulated 78 kDa protein are identical. *Molec. Cellular Biochem.* (accepted).

SCIANDRA, J.J., SUBJECK, J.R. & HUGHES, C.S. (1984). Induction of glucose-regulated proteins during anaerobic exposure and of heat-shock proteins after reoxygenation. *Proc. Natl Acad. Sci. USA*, **81**, 4843.

SUBJECK, J.R. & SHYY, T.-T. (1986). Stress protein systems of mammalian cells. *Am. J. Physiol.*, **19**, C1.

SUTHERLAND, R.M. (1988). Cell and environment interactions in tumor microregions: the multicell spheroid model. *Science*, **240**, 117.

VON SONNTAG, C. (1987). Protection, sensitization and the oxygen effect. In: *The Chemical Basis of Radiation Biology*, p. 295. Taylor and Francis, Inc.: New York.

WILSON, R.E., KENG, P.C. & SUTHERLAND, R.M. (1989). Drug resistance in chinese hamster ovary cells during recovery from severe hypoxia. *J. Natl Cancer Inst.*, **81**, 1235.

WILSON, R.E. & SUTHERLAND, R.M. (1989). Enhanced synthesis of specific proteins, RNA, and DNA caused by hypoxia and reoxygenation. *Int. J. Radiat. Oncol. Biol. Phys.*, **16**, 957.

YOSHIDA, T., BIRO, B., COHEN, T., MULLER, R.M. & SHIBAHARA, S. (1988). Human heme oxygenase cDNA and induction of its mRNA by hemin. *Eur. J. Biochem.*, **171**, 457.